# Adversarial denoising of EEG signals: a comparative analysis of standard GAN and WGAN-GP approaches

**DOI:** 10.3389/fnhum.2025.1583342

**Published:** 2025-05-06

**Authors:** Imad Eddine Tibermacine, Samuele Russo, Francesco Citeroni, Giuseppe Mancini, Abdelaziz Rabehi, Amal H. Alharbi, El-Sayed M. El-kenawy, Christian Napoli

**Affiliations:** ^1^Department of Computer, Automation and Management Engineering, Sapienza University of Rome, Rome, Italy; ^2^Department of Psychology, Sapienza University of Rome, Rome, Italy; ^3^Telecommunications and Smart Systems Laboratory, University of Djelfa, Djelfa, Algeria; ^4^Department of Computer Sciences, College of Computer and Information Sciences, Princess Nourah bint Abdulrahman University, Riyadh, Saudi Arabia; ^5^School of ICT, Faculty of Engineering, Design and Information & Communications Technology (EDICT), Bahrain Polytechnic, Isa Town, Bahrain; ^6^Applied Science Research Center. Applied Science Private University, Amman, Jordan; ^7^Institute for Systems Analysis and Computer Science, Italian National Research Council, Rome, Italy; ^8^Department of Computational Intelligence, Czestochowa University of Technology, Czestochowa, Poland

**Keywords:** EEG denoising, generative adversarial network, Wasserstein GAN, brain-computer interface, deep learning

## Abstract

**Introduction:**

Electroencephalography (EEG) signals frequently contain substantial noise and interference, which can obscure clinically and scientifically relevant features. Traditional denoising approaches, such as linear filtering or wavelet thresholding, often struggle with nonlinear or time-varying artifacts. In response, the present study explores a Generative Adversarial Network (GAN) framework to enhance EEG signal quality, focusing on two variants: a conventional GAN model and a Wasserstein GAN with Gradient Penalty (WGAN-GP).

**Methods:**

Data were obtained from two distinct EEG datasets: a “healthy” set of 64-channel recordings collected during various motor/imagery tasks, and an “unhealthy” set of 18-channel recordings from individuals with orthopedic impairments. Both datasets underwent comprehensive preprocessing, including band-pass filtering (8–30 Hz), channel standardization, and artifact trimming. The training stage involved adversarial learning, in which a generator sought to reconstruct clean EEG signals while a discriminator (or critic in the case of WGAN-GP) attempted to distinguish between real and generated signals. The model evaluation was conducted using quantitative metrics such as signal-to-noise ratio (SNR), peak signal-to-noise ratio (PSNR), correlation coefficient, mutual information, and dynamic time warping (DTW) distance.

**Results:**

Experimental findings indicate that adversarial learning substantially improves EEG signal fidelity across multiple quantitative metrics. Specifically, WGAN-GP achieved an SNR of up to 14.47 dB (compared to 12.37 dB for the standard GAN) and exhibited greater training stability, as evidenced by consistently lower relative root mean squared error (RRMSE) values. In contrast, the conventional GAN model excelled in preserving finer signal details, reflected in a PSNR of 19.28 dB and a correlation coefficient exceeding 0.90 in several recordings. Both adversarial frameworks outperformed classical wavelet-based thresholding and linear filtering methods, demonstrating superior adaptability to nonlinear distortions and dynamic interference patterns in EEG time-series data.

**Discussion:**

By systematically comparing standard GAN and WGAN-GP architectures, this study highlights a practical trade-off between aggressive noise suppression and high-fidelity signal reconstruction. The demonstrated improvements in signal quality underscore the promise of adversarially trained models for applications ranging from basic neuroscience research to real-time brain–computer interfaces (BCIs) in clinical or consumer-grade settings. The results further suggest that GAN-based frameworks can be easily scaled to next-generation wireless networks and complex electrophysiological datasets, offering robust and dynamic solutions to long-standing challenges in EEG denoising.

## 1 Introduction

Linear methods for signal enhancement (Weinstein et al., 1994), such as least-mean-square (LMS) algorithms and their variants (Benesty et al., 2017), are commonly used for noise reduction due to their simplicity (Corino et al., 2006). However, their effectiveness is limited when dealing with non-linear signals, and they often fail to reach a global optimum to eliminate noise and interference (Garrett et al., 2003; Boutarfaia et al., 2023). In contrast, nonlinear techniques Gourévitch et al. (2006), such as wavelet transform with adaptive thresholding, leverage strong local time-frequency analysis (Abramovich and Benjamini, 1996; Nasri and Nezamabadi-pour, 2009; Tibermacine et al., 2024b) to remove non-stationary noise Rabbani et al. (2011); Krishnaveni et al. (2006).

Deep neural networks (DNNs) (Sze et al., 2017) extend beyond traditional filtering by learning complex patterns of noise and interference (Sun et al., 2018), making them suitable for environments with substantial interference and dynamic behavior (Mao et al., 2018; Russo et al., 2024b). Auto-encoders, in particular, have demonstrated effectiveness for noise reduction (O'Shea et al., 2016; Feng et al., 2017; Nail et al., 2024). However, the unpredictable nature of real-world channels—wireless or otherwise—can degrade fixed or purely data-driven enhancement strategies (Simsek et al., 2014; Tibermacine et al., 2023; Gannot et al., 2001; Ladjal et al., 2024).

A major breakthrough in generative modeling came with Generative Adversarial Networks (GANs) (Goodfellow et al., 2014), known for their ability to learn complex data distributions in a minimally supervised fashion. Subsequent innovations, such as the Wasserstein GAN (WGAN) (Arjovsky et al., 2017) and its gradient-penalty variant (Gulrajani et al., 2017), have further stabilized training and improved generation quality, which is crucial when preserving subtle signal characteristics.

Motivated by these developments, researchers have applied GAN-based frameworks to EEG denoising tasks, demonstrating resilience to dynamic noise patterns. Zhou et al. (2020) introduced WSE-GAN, primarily for wireless signals, yet conceptually relevant to non-stationary EEG (Bouchelaghem et al., 2024; Tibermacine et al., 2024c). Luo et al. (2020); Naidji et al. (2023) proposed a WGAN with Temporal-Spatial-Frequency (TSF) loss to preserve multiple EEG dimensions, though at the cost of computational complexity (Sanei and Chambers, 2013; Wang and Bovik, 2009; Gao et al., 2020). Overall, GAN methods excel at adaptively filtering complex and time-varying interference (Judd et al., 2008; Anguera et al., 2013; Yin et al., 2024), making them particularly attractive for EEG signal reconstruction, where both robustness and efficiency are needed.

While prior studies have shown the promise of GANs for EEG denoising (Goodfellow et al., 2014; Zhou et al., 2020; Luo et al., 2020), there is limited direct comparison of a standard GAN against a WGAN-GP in the same experimental setting. Consequently, our work (i) Examines the trade-off between preserving crucial EEG details and suppressing noise, shedding light on when one model may excel over the other, particularly in clinical scenarios requiring subtle neural signal fidelity versus high-noise environments favoring stronger artifact reduction, (ii) Highlights clear guidelines for selecting an appropriate adversarial framework (GAN vs. WGAN-GP), informed by both quantitative metrics (e.g., signal-to-noise ratio, correlation) and practical considerations (e.g., computational overhead).

By addressing these aspects, our study provides novel insights into how the two adversarial architectures perform under varying artifact conditions and signal demands.

**Our key contributions:**

1. **GAN-based wireless/EEG enhancement:** we design and analyze an adversarial pipeline for noise suppression, demonstrating its ability to handle nonlinear distortions in both wireless and EEG contexts.
2. **Comparison of standard GAN and WGAN-GP:** We comprehensively evaluate their performance in terms of noise suppression *versus* detail retention, offering nuanced guidance on the use-case scenarios for each.
3. **Robust evaluation on a dedicated EEG dataset:** our experiments apply multiple signal-quality metrics, underscoring how adversarial learning can be readily extended to broader signal processing challenges.
4. **Implications for clinical and high-interference settings:** our results delineate how each model's strengths or weaknesses suit clinical (low tolerance for signal distortion) or high-interference (priority on aggressive artifact rejection) conditions, thus clarifying deployment strategies in real-world scenarios.

## 2 Related works

Traditional EEG denoising has frequently relied on signal processing approaches such as filtering, regression, and wavelet-based thresholding. Although these methods are well-suited for stationary or linear types of noise, they often underperform when tasked with removing complex, nonlinear EEG artifacts (e.g., ocular or muscle noise). Such limitations have led researchers to explore more sophisticated, data-driven approaches involving deep neural networks and, increasingly, GANs Goodfellow et al. (2014); Arjovsky et al. (2017); Gulrajani et al. (2017).

### 2.1 GAN- and WGAN-based methods

Zhou et al. (2020) introduced a Wireless Signal Enhancement GAN (WSE-GAN) to suppress channel noise in simulated wireless data. Although developed primarily for communication signals rather than EEG, their framework underscored the ability of adversarial models to filter dynamic, non-stationary noise. Focusing directly on EEG, Luo et al. (2020) proposed a WGAN with temporal–spatial–frequency (TSF) loss to better preserve the multi-dimensional structure of neural signals (Russo et al., 2024a). By integrating spectral, spatial, and temporal aspects of EEG, WGAN-TSF achieved strong artifact suppression as well as better classification outcomes in downstream motor-imagery tasks. Subsequently, Zhang et al. employed a WGAN-GP to devise an *Artifact Removal WGAN (AR-WGAN)*, which outperformed several classical denoising benchmarks in both correlation and error metrics (Zhang et al., 2021; Tibermacine et al., 2024a), though over-suppression of low-frequency components was occasionally observed in heavily contaminated inputs.

### 2.2 Conditional and task-specific GANs

Kim et al. (2022) presented a conditional GAN (cGAN) framework tailored to remove multiple classes of EEG artifacts (e.g., ocular or muscle noise) by conditioning on artifact-related labels or signal features. This approach yielded significant improvements in SNR and correlation with ground-truth signals compared to conventional filters, illustrating the advantage of leveraging context during artifact removal.

While these works highlight the adaptability and strength of adversarial learning in mitigating complex EEG artifacts, they leave open several questions. For instance, direct comparisons among *vanilla* GAN, WGAN (with or without GP), and classical methods under identical conditions remain rare. In addition, most studies focus on limited subsets of metrics, making it difficult to evaluate trade-offs between artifact removal effectiveness and signal fidelity. Table 1 presents a concise overview of major prior GAN-based EEG denoising approaches.

**Table 1:** Representative studies on adversarial approaches for EEG denoising.

**References**	**Model**	**Dataset**	**Evaluation metrics**	**Key findings**
Zhou et al. (2020)	WSE-GAN (standard GAN)	Simulated wireless signals	SNR, BER, interference levels	Demonstrated that GAN-based modeling can adaptively filter dynamic noise and interference.
Luo et al. (2020)	WGAN + TSF loss	Public EEG datasets (motor imagery)	MSE, classification accuracy	Improved artifact removal across temporal, spatial, and frequency dimensions; boosted BCI performance.
Zhang et al. (2021)	AR-WGAN (WGAN-GP)	EEGdenoiseNet + self-collected EEG	Correlation, RRMSE, PSD	Outperformed classical baselines with high correlation and low error, but risked over-suppressing low-frequency content.
Kim et al. (2022)	cGAN (conditional GAN)	EEG with ocular/muscle artifacts	SNR, MAE, correlation	Successfully targeted multiple artifact types using contextual information; retained high neural signal fidelity.

The literature confirms that adversarial networks—in their various forms—offer compelling solutions for EEG artifact removal, often surpassing classical denoising filters. However, explicit head-to-head comparisons between a *standard* GAN and a *WGAN-based* model, alongside classic baseline methods, have not been thoroughly reported. Moreover, certain practical issues, such as computational overhead, the role of gradient penalties, and performance across diverse EEG datasets, remain insufficiently investigated. The present work addresses these gaps by comprehensively comparing a vanilla GAN and a WGAN-based architecture (both with and without gradient penalty) to classical denoising methods, all under the same experimental design and evaluation framework.

## 3 Materials and methods

### 3.1 Datasets

#### 3.1.1 Healthy dataset

This subset comprises 64-channel EEG recordings collected from 109 volunteers, yielding over 1,500 individual recordings of one to two minutes each. The dataset is publicly available (Schalk et al., 2022), and all data were acquired using the BCI2000 platform.^1^ Each participant completed 14 experimental runs, including two baseline runs (one with eyes open and one with eyes closed) and three two-minute runs of each of the following four tasks:

1. **Task 1:** A target appears on the left or right side of the screen, prompting the participant to repeatedly open and close the corresponding fist until the target disappears, followed by rest.
2. **Task 2:** Similar to Task 1, but the participant *imagines* the corresponding fist movements rather than physically executing them.
3. **Task 3:** A target at the top or bottom of the screen instructs opening/closing of both fists (top) or both feet (bottom), ending with rest.
4. **Task 4:** Identical to Task 3 except that the movements are *imagined* rather than physically performed.

Each event is labeled with an event-type indicator (T0, T1, or T2) concatenated with a task identifier (e.g., TASK1T2). These labels specify rest intervals (T0) or onsets of physical/imagined motions (T1, T2). Table 2 outlines the key characteristics of this dataset.

**Table 2:** Overview of the Healthy Dataset.

**Item**	**Description**	**Notes**
Number of channels	64	Standard scalp montage
Number of participants	109	Volunteers with motor/imagery tasks
Total recordings	>1,500	1–2 minutes each
Acquisition platform	BCI2000	Open/closed eyes baseline + tasks
Number of tasks	4	Physical/Imagined: fists or feet
Event codes	T0, T1, T2	Context-dependent labeling
Baseline runs	2 (Eyes open/closed)	1 minute each
Task runs per task	3 (Each of four tasks)	2 minutes each

#### 3.1.2 Unhealthy dataset:

A second dataset contains raw 18-channel EEG signals (Table 3) obtained from seven participants with orthopedic impairments during motor-imagery (MI) tasks (Lee et al., 2022). Data collection occurred in three sessions, each comprising 40 trials, and included four distinct MI tasks presented in a randomized sequence (e.g., *Reach* → *Twist* → *Lift* → *Grasp*, etc.). Each trial started with a 3 s fixation cross, followed by a 4 s visual cue and a 3 s transition (“ready”) period, culminating in a 5 s imagined movement. This design gathered motor-imagery data from both healthy and impaired populations, broadening the scope for robust denoising analysis.

**Table 3:** Overview of the unhealthy dataset.

**Item**	**Description**	**Notes**
Number of channels	18	Orthopedic impairment study
Number of participants	7	Motor-imagery with physical constraints
Sessions per participant	3	Each session: 40 trials
Trials per session	40	Randomized MI tasks
MI tasks	4	e.g., Reach, Twist, Lift, Grasp
Trial structure	3s fixation, 4s cue, 3s ready, 5s MI	Total ~15s per trial

#### 3.1.3 Dataset variability and robustness

To ensure that random sample selection does not omit important sources of variability, our experiments utilized *all available* recordings from both the healthy and unhealthy datasets. For each denoising approach (GAN, WGAN-NoGP, WGAN-GP, and classical baselines), we computed and reported *aggregate* performance metrics over the complete set of EEG sessions (where practical). In addition, we provide *mean* ± *standard deviation* for key measures (SNR, MAE, etc.), offering insight into the consistency of denoising outcomes across participants, channels, and trials. This comprehensive evaluation helps confirm the generalizability of our findings, especially given the diversity of tasks (physical vs. imagined movements) and participant conditions (healthy vs. orthopedic impairments).

### 3.2 Preprocessing

A multi-step preprocessing pipeline was employed to ensure data consistency, quality, and relevance for subsequent analysis. First, each comma-separated values (CSV) file was aligned with a reference tab-separated values (TSV) file, thereby standardizing channel labels and headers across all recordings. Data were then filtered according to task labels: for the unhealthy dataset, only rows labeled “S 1,” “S 4,” “S 8,” or “S 10” were retained, while for the healthy dataset, only rows labeled “TASK1T1” or “TASK1T2” were included. Columns determined to be nonessential (e.g., the final label column) were removed to streamline the dataset.

Next, any channels not shared between healthy and unhealthy datasets were discarded, ensuring uniform channel availability for comparative analysis. The resulting dataframes were then normalized to the range [0, 1] via Min–Max scaling, mitigating the influence of disproportionate feature ranges. We focused on the sensorimotor cortex, thus retaining only channels named C3, C4, Cz (and any variants with prefix “C”) from the standard 10–20 montage.

Finally, each EEG data tensor was inspected to confirm that it met the minimum required shape. Any tensors smaller than this threshold were resized or cropped, thereby maintaining consistent dimensionality. This integrated workflow produced a coherent, high-quality dataset, suitably prepared for the subsequent modeling and analysis phases.

### 3.3 Band pass filter

To enhance the quality of the EEG signals and isolate relevant frequency components, a bandpass filter is applied to the dataset. The filtering process is crucial for removing unwanted noise and focusing on the specific frequency range of interest. In this study, a bandpass filter with a passband of 8–30 Hz is employed, which is commonly used to target the alpha and beta frequency bands in EEG analysis.

The filtering procedure begins by extracting channel names from the first row of each DataFrame. The DataFrame is then converted into an MNE Raw object, which is a data structure specifically designed for handling EEG data. Using the MNE-Python library, the bandpass filter is applied to the Raw object. This step involves specifying the low and high cutoff frequencies (8–30 Hz, respectively) and the sampling frequency (128 Hz).

After filtering, the channel names are reinserted into the DataFrame, and the filtered data are extracted from the Raw object. The data is then converted back into a DataFrame format with the original channel names. This ensures that the filtered data maintains the same structure as the input DataFrames.

The bandpass filter is applied to both unhealthy and healthy datasets, refining the signals and preparing them for further analysis. This preprocessing step is essential to improve signal quality and improve the accuracy of subsequent modeling and analysis phases.

## 4 Model

**Figure 1:**
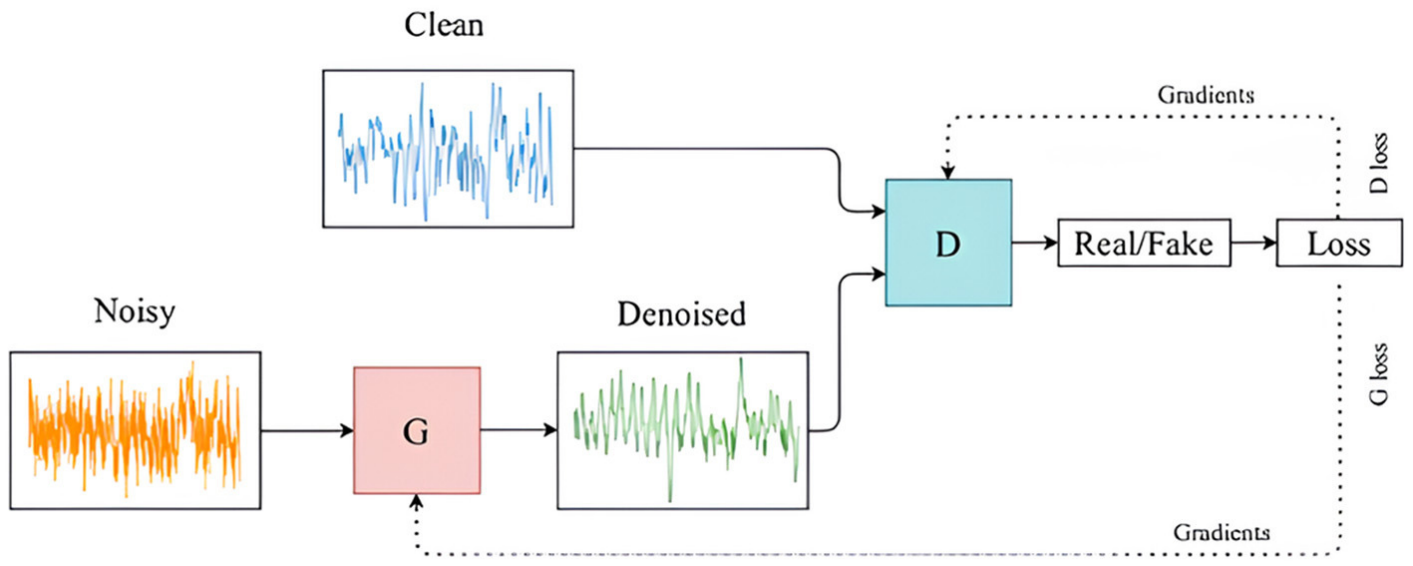
GANArchitecture for converting noisy EEG signals to clean EEG data.

**Figure 2:**
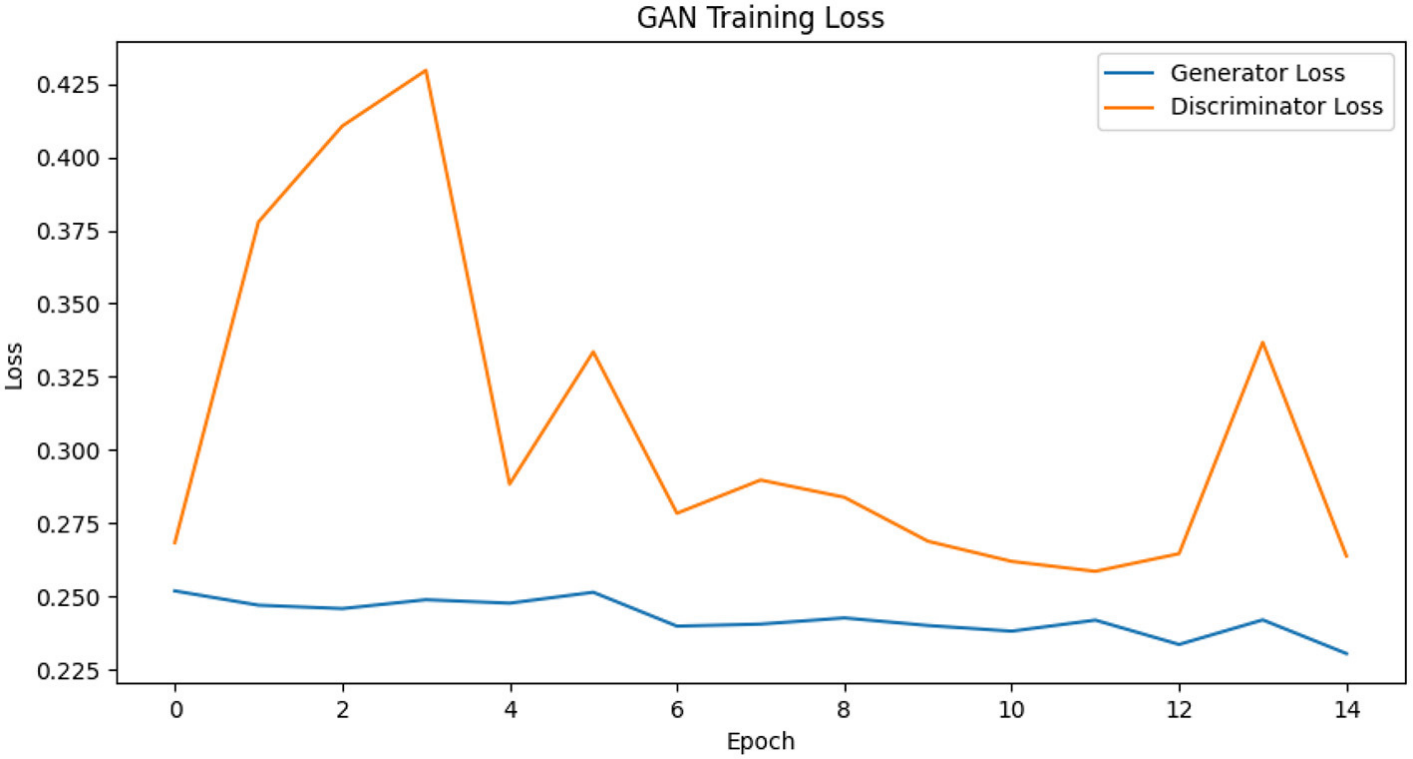
GANTraining loss.

GANs are a type of generative model that is used to create synthetic data without requiring detailed domain-specific knowledge. They were introduced by Goodfellow et al. (2014), in which a multi-layer perceptron was employed for both the generator and the discriminator networks. These two networks engage in a competitive process, often referred to as a minimax game, as described by the objective function in Equation 1. The generator aims to increase the error rate of the discriminator by producing data that resemble real samples, while the discriminator tries to accurately differentiate between real and generated samples. This interaction is illustrated in Figures 1, 2. GANs are widely applied to generate new, previously unseen data, either for augmenting existing datasets or to ensure the privacy of the training data.


(1) $$\begin{array}{r} \begin{array}{r} \underset{G}{\min}\underset{D}{\max}V\left(G,D\right)={𝔼}_{x\sim p_{data}\left(x\right)}\left[\log D\left(x\right)\right]\\ +{𝔼}_{z\sim p_{z}\left(z\right)}\left[\log\left(1-D\left(G\left(z\right)\right)\right)\right] \end{array} \end{array}$$

### 4.1 EEG signal reconstruction model–LSTM layer

The first Generator implementation was a neural network-based model for EEG signal reconstruction, which utilizes a Long Short-Term Memory (LSTM) layer to model the temporal dependencies in the signal. The model architecture is designed to process sequential EEG data. It consists of an LSTM layer followed by a fully connected (linear) layer, with an optional Tanh activation function for output regularization.

The LSTM layer is the core component for learning temporal dependencies in EEG signals. Given an input sequence *X* = {*x*_1_, *x*_2_, …, *x*_*T*_} where *x*_*t*_ represents the input at timestep *t*, the LSTM produces hidden states *h*_*t*_ and cell states *c*_*t*_ at each timestep. The LSTM's internal operations are governed by the following equations:


(2) $$\begin{array}{l} f_{t}=\sigma \left(W_{f}x_{t}+U_{f}h_{t-1}+b_{f}\right)\;\text{(forget gate)} \end{array}$$

(3) $$\begin{array}{l} i_{t}=\sigma \left(W_{i}x_{t}+U_{i}h_{t-1}+b_{i}\right)\;\text{(input gate)} \end{array}$$

(4) $$\begin{array}{l} {\tilde{c}}_{t}=\tanh\left(W_{c}x_{t}+U_{c}h_{t-1}+b_{c}\right)\;\text{(candidate cell state)} \end{array}$$

(5) $$\begin{array}{l} c_{t}=f_{t}⊙c_{t-1}+i_{t}⊙{\tilde{c}}_{t}\;\text{(cell state update)} \end{array}$$

(6) $$\begin{array}{l} o_{t}=\sigma \left(W_{o}x_{t}+U_{o}h_{t-1}+b_{o}\right)\;\text{(output gate)} \end{array}$$

(7) $$\begin{array}{l} h_{t}=o_{t}⊙\tanh\left(c_{t}\right)\;\text{(hidden state)} \end{array}$$

Here, σ is the Sigmoid function, ⊙ denotes element-wise multiplication, and *W*, *U*, and *b* are the learnable weight matrices and biases for each gate. The LSTM is initialized with zero-valued hidden states and cell states, *h*_0_ and *c*_0_, for each sequence.

Once the LSTM processes the entire sequence, the output is passed through a fully connected (FC) layer to map the hidden state at each timestep *h*_*t*_ to the desired output size *y*_*t*_, represented as:


(8) $$\begin{array}{l} y_{t}=W_{fc}h_{t}+b_{fc} \end{array}$$

where *W*_*fc*_ and *b*_*fc*_ are the weights and biases of the FC layer. This operation converts the LSTM's output to a format suitable for signal reconstruction.

The Sigmoid activation function is applied to the output layer to constrain the output to a specific range (e.g., for normalized EEG signals):


(9) $$\begin{array}{l} y_{t}^{\prime }=\sigma \left(y_{t}\right) \end{array}$$

where $\sigma \left(x\right)=\frac{1}{1+e^{-x}}$ is the sigmoid function. This ensures that the model output is bounded between 0 and 1, which is useful for many signal processing applications.

The combination of the ability of the LSTM to capture long-term dependencies and the flexibility of the fully connected layer makes this model highly suitable for EEG signal reconstruction tasks, where capturing short- and long-term temporal patterns is crucial for accurate performance.

### 4.2 EEG Signal reconstruction model—residual neural network

The second Generator implementation was designed to transform the input sequence into a refined output using a series of convolutional and deconvolutional layers with intermediate residual blocks to enhance feature representation. The network begins with an initial convolutional layer, which applies a 1D convolution with a kernel size of 3 and 64 filters, followed by a batch normalization layer to standardize the output. The activation function used is the rectified linear unit (ReLU), which is defined as:


(10) $$\begin{array}{l} \text{ReLU}\left(x\right)=\max\left(0,x\right) \end{array}$$

This activation function ensures that non-negative inputs pass through while negative values are set to zero, allowing for non-linearity in the model. The output of the first convolutional layer can be represented as:


(11) $$\begin{array}{l} y_{1}=\text{ReLU}\left(\text{BN}\left(\text{Conv1D}\left(x\right)\right)\right) \end{array}$$

where BN denotes batch normalization and Conv1D represents the 1D convolution operation.

Following the initial convolutional layer, the generator passes the data through a series of 16 residual blocks. Each residual block consists of two convolutional layers with kernel size 3, batch normalization, and ReLU activations. The primary advantage of residual blocks is the inclusion of skip connections, allowing the input to bypass the convolutional layers and be added directly to the output. This can be mathematically represented as:


(12) $$\begin{array}{l} y_{k+1}=\text{ReLU}\left(y_{k}+\text{Block}\left(y_{k}\right)\right) \end{array}$$

where Block(*y*_*k*_) represents the output of the convolutional block in the *k*-th residual block.

Once the signal passes through the residual blocks, it is processed by a deconvolutional layer, which expands the dimensionality of the sequence. This deconvolution layer uses a transposed convolution operation with a stride of 2, effectively upsampling the data. The formula for the output after the deconvolutional layer is the following.


(13) $$\begin{array}{l} y_{2}=\text{ReLU}\left(\text{BN}\left(\text{ConvTranspose1D}\left(y_{1}\right)\right)\right) \end{array}$$

Finally, the generator outputs the signal using a 1D convolutional layer with a sigmoid activation function, which ensures that the final output is scaled between 0 and 1. Thus, the final output of the generator is as follows:


(14) $$\begin{array}{l} y_{\text{final}}=\sigma \left(\text{Conv1D}\left(y_{2}\right)\right) \end{array}$$

In general, the architecture effectively combines convolutional layers, residual blocks, and deconvolution operations to refine the input signal and produce the desired output. The use of residual connections helps mitigate the vanishing gradient problem, allowing the network to learn more effectively.

### 4.3 Discriminator

The Discriminator model is designed as a deep convolutional neural network (CNN) with the objective of distinguishing real data sequences from those generated by the adversarial model. The input to the Discriminator is a one-dimensional sequence of size *seq_len* with *input_size* features. The model architecture follows a sequence of convolutional layers, each increasing in complexity to progressively extract higher-level features from the input sequence.

The network consists of eight convolutional layers. The first layer applies a convolution with 64 filters, each of size 3, using a stride of 1, followed by batch normalization to stabilize learning and LeakyReLU activation to introduce non-linearity. As the layers progress, the number of filters doubles, while the stride alternates between 1 and 2, which reduces the sequence length. The convolutional operation in each layer can be expressed as:


(15) $$\begin{array}{l} y\left(t\right)=\overset{K}{\underset{k=1}{\sum }}w_{k}\cdot x\left(t-k+1\right)+b \end{array}$$

where *y*(*t*) is the output at the time step *t*, *w*_*k*_ is the convolution filter, *x*(*t* − *k* + 1) represents the input values, and *b* is the bias term. Padding ensures that the output dimensions are maintained in layers with a stride of 1, while layers with stride 2 downsample the input, effectively reducing the temporal resolution of the sequence.

The final layers in the convolutional block output feature maps of size $1024\times \frac{\text{seq\_len}}{16}$. These are flattened and passed through a fully connected layer to produce a scalar value and at the end was applied a sigmoid function.

The architecture is optimized for binary classification, where the goal is to correctly classify real and generated sequences. By progressively reducing the sequence length while expanding the feature space through convolutions, the Discriminator captures essential temporal dependencies and feature hierarchies crucial for distinguishing between real and generated data.

## 5 EEG-GAN

### 5.1 Loss functions and training setup

In this GAN, both networks are trained with specific loss functions tailored to improve the denoising performance:

1. **Generator loss (content loss)**: the primary objective for the Generator is to create denoised EEG signals that closely match the real, clean EEG data. We employ a Mean Squared Error (MSE) loss function, which measures the difference between the Generator's denoised output and the real, clean EEG signal. The MSE content loss is defined as:
  (16) $$\begin{array}{l} L_{G}=\text{MSE}\left(\text{Generator}\left(x_{\text{noisy}}\right),x_{\text{real}}\right) \end{array}$$
  where *x*_noisy_ represents the input noisy EEG, and *x*_real_ is the ground truth clean EEG signal. This loss ensures that the Generator output is as close as possible to the real EEG data in terms of amplitude and temporal structure.
2. **Discriminator loss (adversarial loss)**: the Discriminator is trained to classify EEG samples as real (clean EEG) or fake (denoised output from the Generator). We use a combination of real and fake labels with the MSE loss to compute the Discriminator's loss. The Discriminator loss is computed as:
  (17) $$\begin{array}{l} L_{D}=\frac{1}{2}\left(\begin{array}{r} \text{MSE}\left(D\left(x_{\text{real}}\right),y_{\text{real}}\right)\\ +\text{MSE}\left(D\left(\text{Generator}\left(x_{\text{noisy}}\right)\right),y_{\text{fake}}\right) \end{array}\right) \end{array}$$
  where *y*_real_ = 1 and *y*_fake_ = 0. The Discriminator loss encourages it to correctly classify clean EEG data as real and Generator outputs as fake, thereby refining the Generator's outputs through adversarial training.

Although the original GAN formulation adopts a binary cross-entropy (BCE) loss for the discriminator (Goodfellow et al., 2014), we instead use a mean squared error (MSE) term, following the Least Squares GAN (LSGAN) framework (Mao et al., 2017). By replacing the log-likelihood objective with a least-squares objective, we alleviate issues such as gradient saturation and stabilize training. Concretely, instead of minimizing −log *D*(·) or −log(1 − *D*(·)), we penalize the squared difference from the real or fake label, which empirically yields smoother and more reliable gradient updates in our EEG denoising setting.

In addition to this MSE-based adversarial term, our generator also includes a mean squared error (MSE) (Table 4) *content* or *reconstruction* component. Although we do not explicitly condition on class labels (as in a classic cGAN), this combined objective helps the generator fulfill two complementary goals: (i) producing EEG signals that fool the discriminator (adversarial realism), and (ii) preserving fidelity to the clean reference waveforms (reconstruction accuracy). In practice, this hybrid loss structure preserves subtle temporal and amplitude features of the EEG signal, while still removing artifacts. As a result, it offers a practical safeguard against potential distortions or mode collapse that might arise from a purely adversarial objective.

**Table 4:** GAN training hyperparameters.

**Hyperparameter**	**Value**	**Description**
Optimizer	Adam	Generator and Discriminator optimizer.
Generator LR	0.00002	Learning rate for the generator's optimizer.
Discriminator LR	0.002	Learning rate for the discriminator's optimizer.
Betas	0.5 - 0.999	The beta parameters of the optimizer (momentum term and decay).
Batch Size	32	Number of EEG signals per training batch.
Epochs	15	Number of complete passes through the dataset.
Loss function	Mean Squared Error (MSE)	Content loss function used to measure signal reconstruction accuracy.

### 5.2 Training procedure

The training alternates between updating the Generator and the Discriminator to achieve balanced adversarial dynamics. The Generator is optimized to minimize the content loss by generating denoised EEG signals that match the real EEG data, while the Discriminator is optimized to minimize the adversarial loss by correctly identifying real versus generated signals. The following steps summarize each iteration:

1. The Generator takes a batch of noisy EEG signals and produces a denoised output.
2. The Discriminator evaluates both the real clean EEG data and the generated EEG, computing the adversarial loss for each.
3. The Generator's parameters are updated based on the content loss, while the Discriminator's parameters are updated based on the adversarial loss.

**Algorithm 1:**
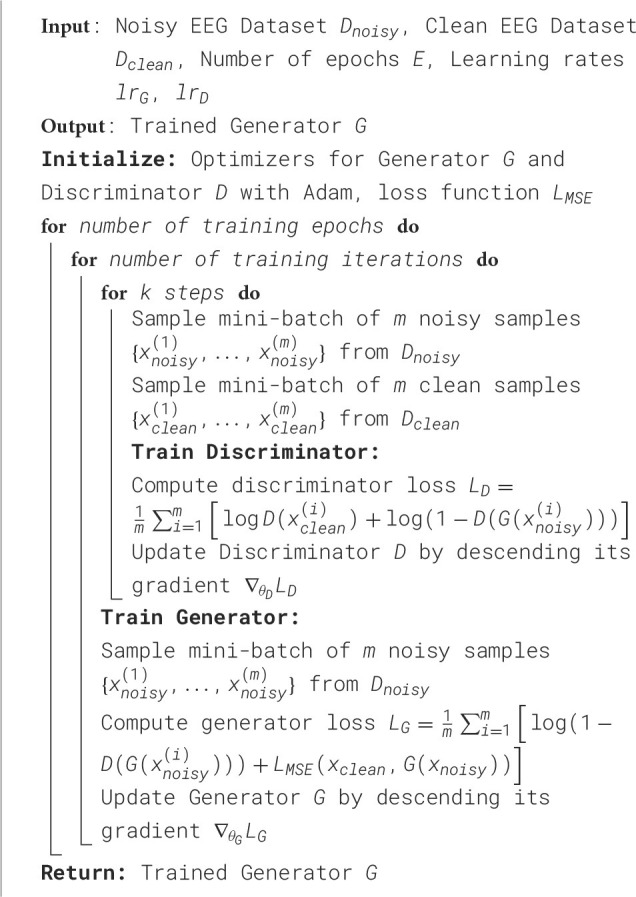
Minibatch stochastic gradient descent training of EEG-GAN for denoising EEG signals.

## 6 Wasserstein generative adversarial network with gradient penalty

To address the challenge of denoising EEG signals, we employ a state-of-the-art WGAN-GP. This architecture leverages adversarial training between a Generator and a Discriminator, ensuring that the generated EEG signals closely resemble clean, healthy EEG data. WGAN-GP provides stability in training by replacing the original GAN's Jensen-Shannon divergence with the Wasserstein distance, which offers smoother gradients and mitigates issues such as mode collapse.

### 6.1 Architecture Design

The WGAN-GP model consists of two core neural networks:

- **Generator**: This network learns to transform noisy EEG inputs into denoised counterparts. It takes as input a 3-dimensional tensor representing the noisy EEG signals and produces a denoised output with the same dimensions. Each layer of the Generator captures temporal dependencies within the EEG signals, enhancing their signal fidelity through upsampling.
- **Discriminator (critic)**: Acting as a surrogate for the Wasserstein distance, the Discriminator aims to differentiate between real clean EEG samples and those generated by the Generator. It outputs a scalar value representing the "realness" of the input, where higher values indicate a closer resemblance to real data.

### 6.2 Training setup and loss functions

The training process alternates between updating the Discriminator and the Generator, as follows:

1. **Discriminator loss**: the Discriminator is trained to maximize the Wasserstein distance between real and fake EEG distributions. This is achieved through the following objective function:
  (18) $$\begin{array}{l} L_{D}=𝔼\left[D\left(x_{\text{real}}\right)\right]-𝔼\left[D\left(x_{\text{fake}}\right)\right]\;+\lambda \cdot 𝔼\left[{\left(∥\nabla D\left(\tilde{x}\right){∥}_{2}-1\right)}^{2}\right] \end{array}$$
  where *D*(*x*_real_) and *D*(*x*_fake_) are the Discriminator outputs for real and generated samples, respectively, and $\tilde{x}$ denotes interpolated samples for gradient penalty calculation. The gradient penalty term, scaled by the hyperparameter λ, ensures that the Lipschitz constraint is maintained, enhancing training stability.
2. **Generator loss**: the Generator aims to produce denoised EEG signals that maximize the Discriminator's output, corresponding to a higher resemblance to real data. The Generator loss is defined as:
  (19) $$\begin{array}{l} L_{G}=-𝔼\left[D\left(x_{\text{fake}}\right)\right] \end{array}$$
  This formulation directs the generator to maximize the discriminator's response to fake EEG data, driving the generated samples closer to the distribution of clean EEG signals.

### 6.3 Gradient penalty computation

The WGAN-GP incorporates a gradient penalty term to enforce the Lipschitz constraint, which is crucial for maintaining stability during training. The penalty term is computed based on an interpolation between real and fake samples:


(20) $$\begin{array}{l} L_{\text{gp}}=\lambda \cdot 𝔼\left[{\left(∥\nabla D\left(\tilde{x}\right){∥}_{2}-1\right)}^{2}\right] \end{array}$$

where $\tilde{x}=\epsilon \cdot x_{\text{real}}+\left(1-\epsilon \right)\cdot x_{\text{fake}}$, with ϵ ~ Uniform(0, 1). The penalty ensures that the Discriminator's gradients are constrained, aligning them with the 1-Lipschitz condition necessary for Wasserstein GAN training.

### 6.4 Training procedure

In this study, we adopted Adam optimizers for both Generator and Discriminator (or Critic), with the Generator updated once per epoch and the Discriminator updated multiple times per Generator step (*n*_critic_ = 5). After preliminary testing of multiple learning rates (1 × 10^−5^ to 5 × 10^−5^), we settled on 2 × 10^−5^ for its stable convergence on a validation subset. For WGAN-GP runs, the gradient penalty coefficient λ was set to 10, balancing effective gradient control without over-penalizing the Critic. A batch size of 64 was determined from initial evaluations in {16, 32, 64}, providing the best compromise between GPU memory usage and consistent performance. Training generally continued for 25 epochs, monitored via an early stopping criterion to prevent overfitting. EEG signals were normalized channel-wise before each run, and the denoised outputs were periodically examined to confirm artifact removal without undue signal distortion. These final hyperparameter settings, once validated through repeated experimentation, were uniformly applied in the GAN, WGAN-NoGP, and WGAN-GP implementations, ensuring a fair foundation for comparing their effects on EEG signal fidelity. Table 5 provides a concise overview of these final selections.

**Algorithm 2:**
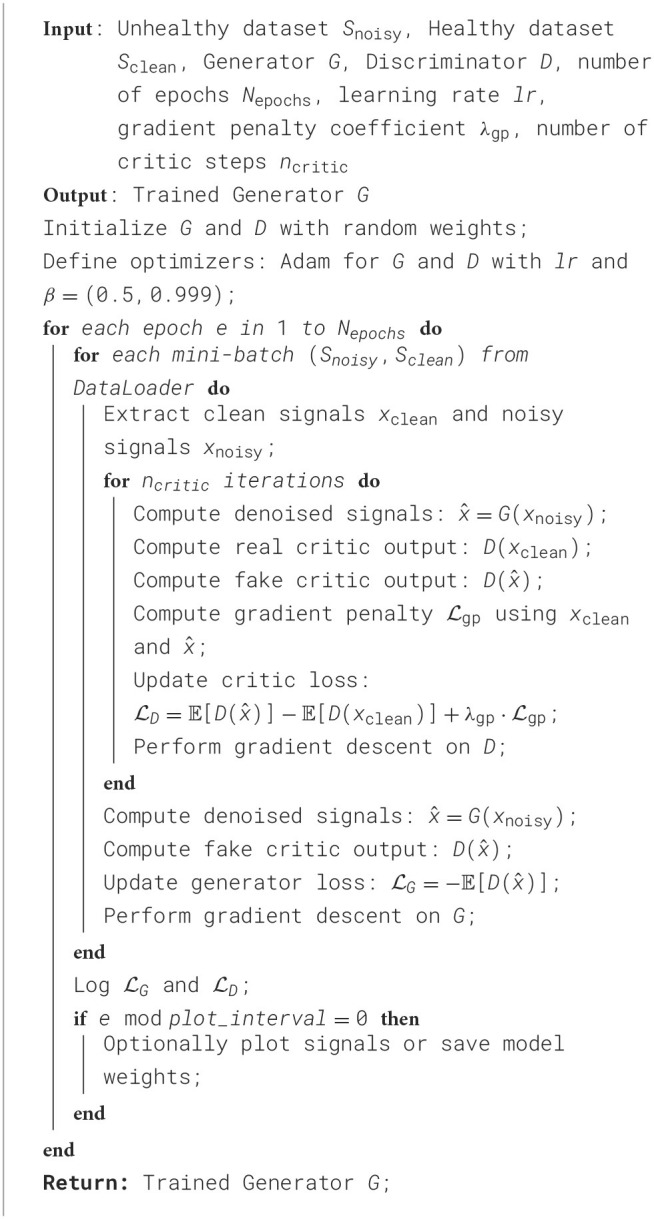
Training of WGAN-GP for EEG Denoising.

**Table 5:** WGAN-GP training hyperparameters.

**Hyperparameter**	**Value**	**Description**
Optimizer	Adam	Generator and Discriminator optimizer.
Generator LR	0.00002	Learning rate for the optimizer.
Discriminator LR	0.00002	Learning rate for the optimizer.
Betas	0.5 - 0.999	The beta parameters of the optimizer (momentum term and decay).
Batch size	64	Number of EEG signals per training batch.
Epochs	25	Number of complete passes through the dataset.
Loss function	Critic Loss	Combines Wasserstein loss with a gradient penalty to enforce Lipschitz continuity.

## 7 Results

### 7.1 Reconstruction results

**Figure 3:**
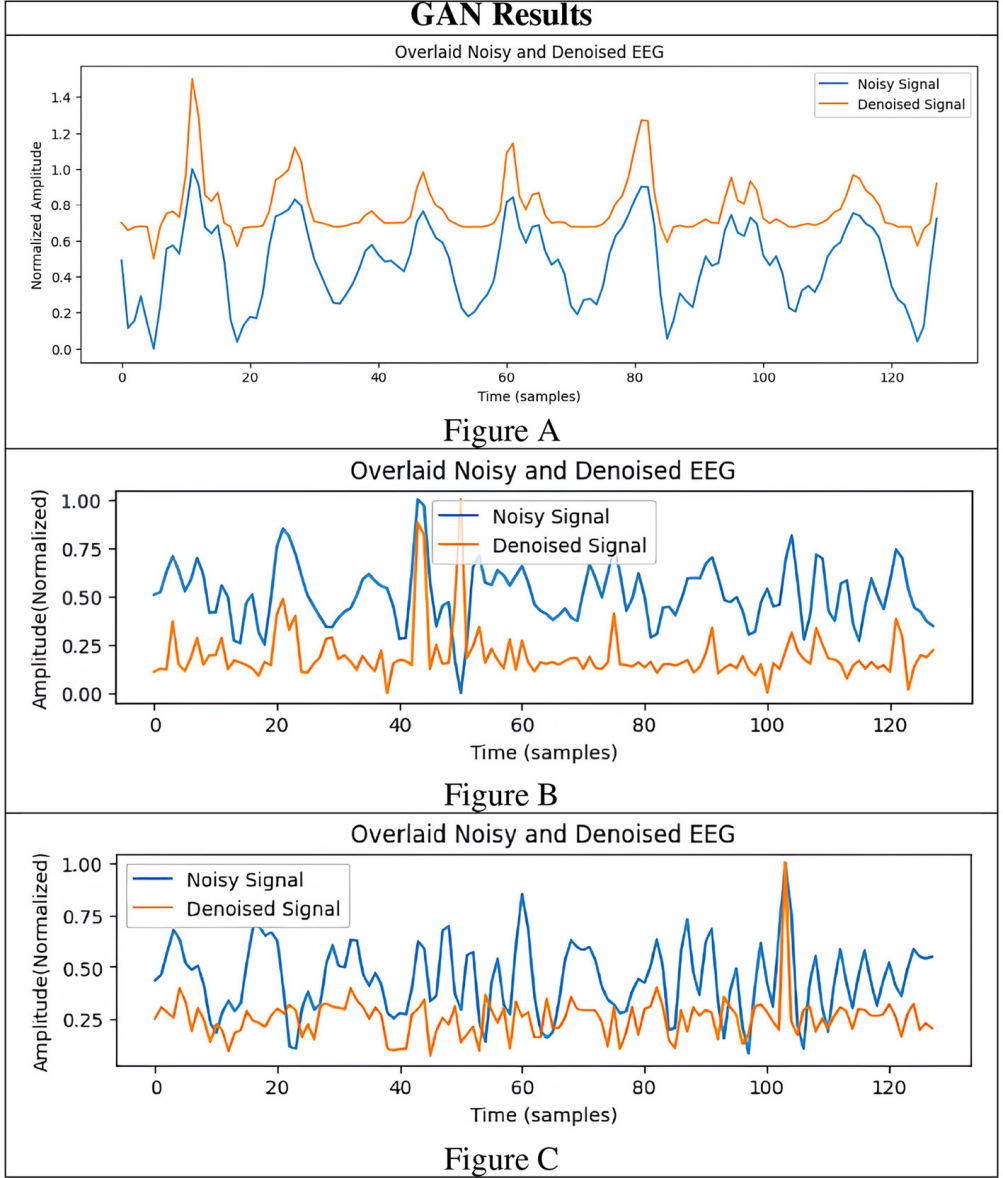
Results of GAN generated EEG signals. **(A–C)** are 3 different result samples.

**Figure 4:**
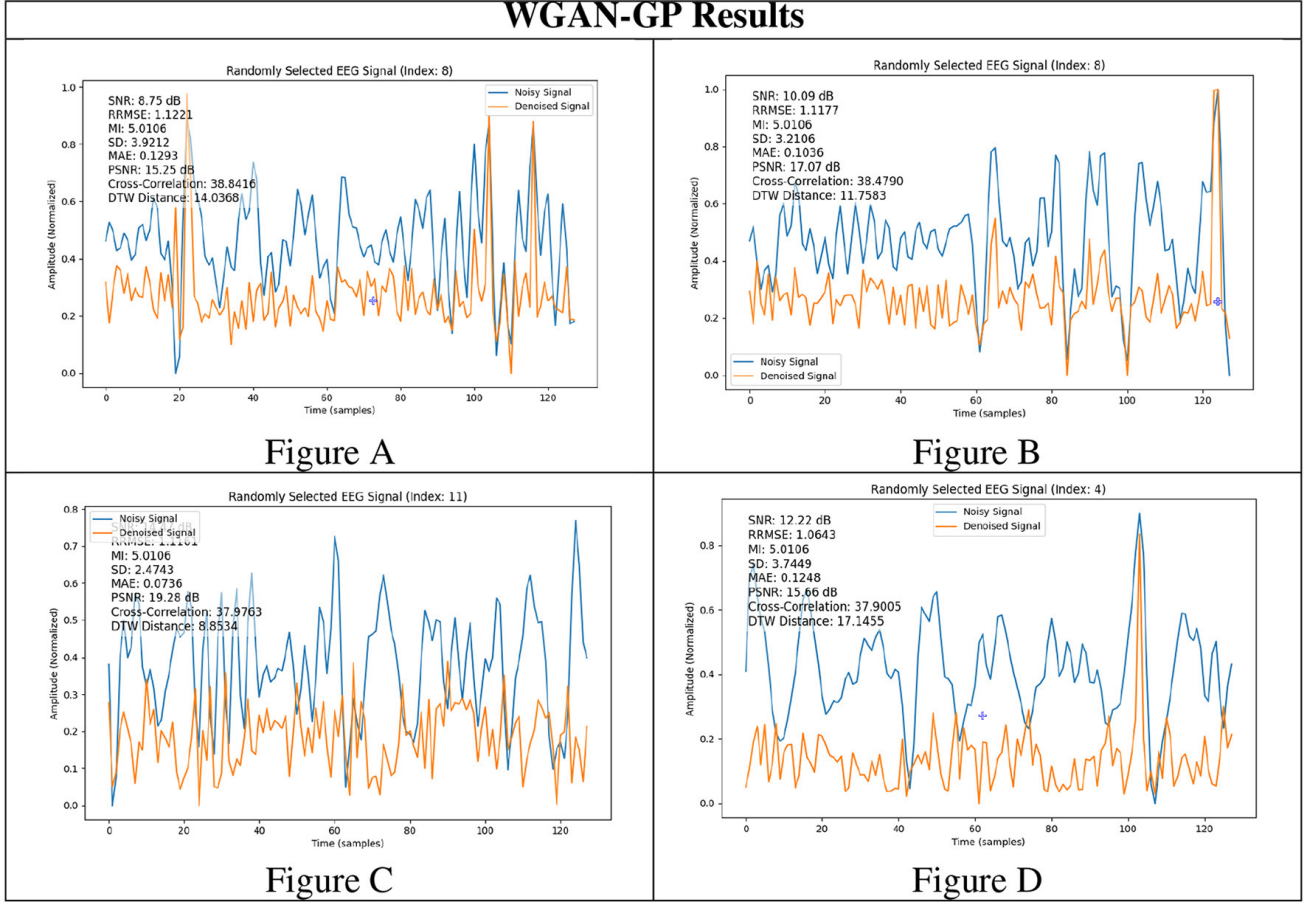
Results of WGAN-GP generated EEG signals. **(A–D)** are 4 different result samples.

To rigorously assess the generalizability of each model, we performed signal reconstructions on EEG channels that were *excluded* during training. This approach ensured that both the EEG-GAN and WGAN-GP architectures were evaluated on data whose characteristics differed from those used to optimize their parameters. As shown in Figures 3A–C (GAN reconstructions) and Figures 4A–C (WGAN-GP reconstructions), both models demonstrated robust performance, indicating minimal overfitting.

To ensure a robust definition of clean EEG signals, we identified low-artifact segments (based on amplitude thresholds and visual inspection) and, where necessary, artificially introduced controlled noise to create noisy-clean paired epochs. This semi-synthetic approach yields a more reliable ground truth for computing metrics such as MAE, SNR, and correlation. Detailed procedures for selecting low-artifact segments and injecting synthetic noise were provided in the *Methods* section, ensuring both transparency and reproducibility.

**Figure 5:**
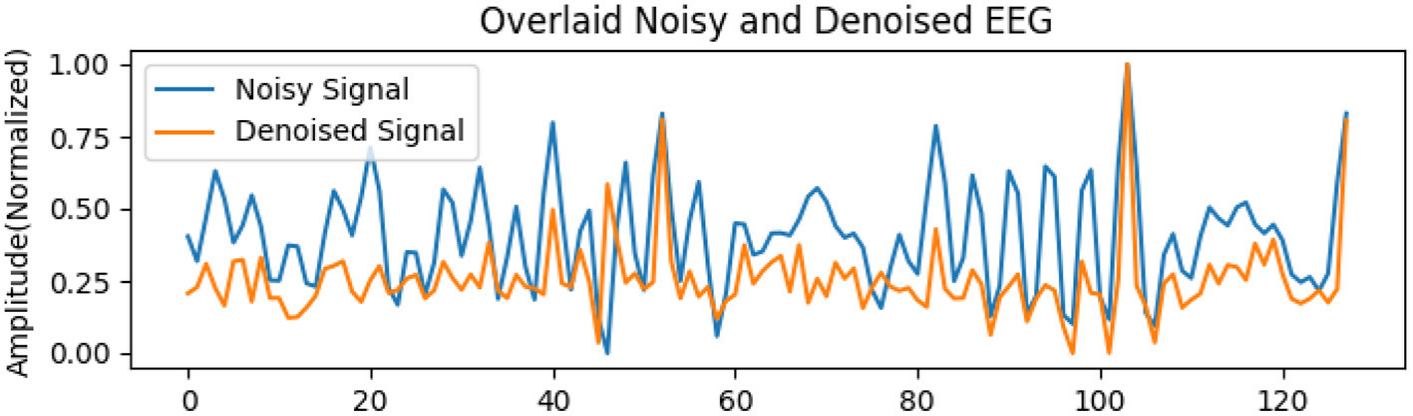
WGAN-GP signal denoising during the training.

**Figure 6:**
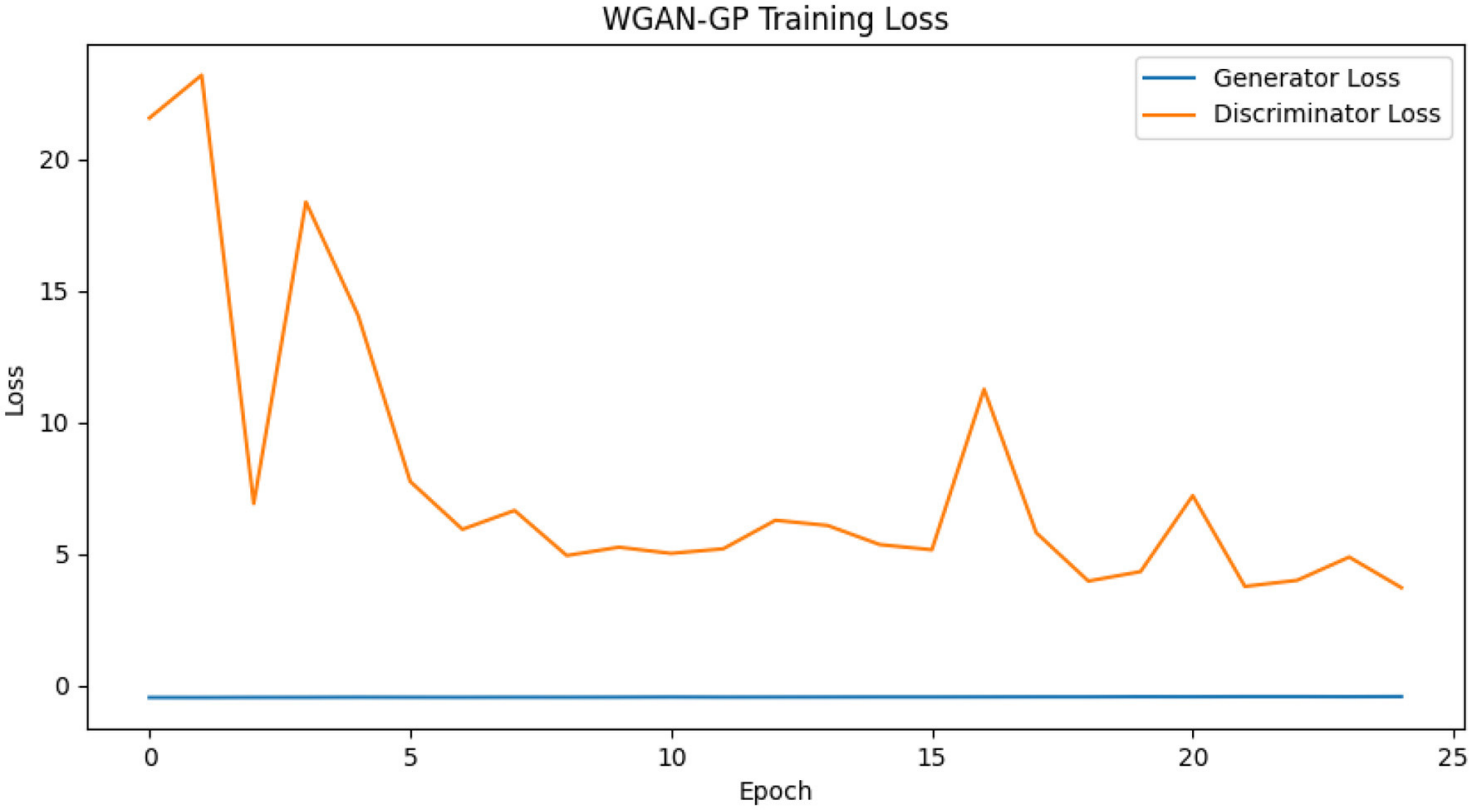
WGAN-GP training loss.

Quantitative metrics, presented in Tables 6 and 7, reveal that the standard GAN often preserves finer details of the signal, as evidenced by the higher PSNR. In contrast, WGAN-GP tended to provide stronger overall noise suppression, reflected in higher SNR values and more stable training dynamics. Taken together, these findings suggest that while EEG-GAN is advantageous in retaining subtle signal features, WGAN-GP excels in scenarios demanding aggressive noise reduction (Figures 5, 6).

**Table 6:** GAN signal denoising evaluation.

**Signal**	**SNR**	**RRMSE**	**MI**	**SD**	**MAE**	**PSNR**	**DTW distance**
Figure 3A	12.37	0.818	4.96	2.33	0.069	19.28	10.42
Figure 3B	11.89	0.974	5.02	3.38	0.090	18.47	11.47
Figure 3C	11.29	1.006	5.08	3.13	0.143	19.26	12.41

**Table 7:** WGAN signal denoising evaluation.

**Signal**	**SNR**	**RRMSE**	**MI**	**SD**	**MAE**	**PSNR**	**DTW distance**
Figure 4A	8.75	1.122	5.01	3.92	0.129	15.25	14.03
Figure 4B	10.09	1.117	5.03	3.21	0.103	17.07	11.75
Figure 4C	14.47	1.116	5.08	2.47	0.073	19.28	8.85
Figure 4D	12.22	1.064	5.06	3.74	0.124	15.66	17.14

In addition, both frameworks successfully distinguished between healthy and unhealthy EEG signals, which underscoring their potential value in various clinical and neuroscientific contexts. Their ability to reconstruct different signal types with minimal degradation highlights the adaptability of adversarial learning to denoise EEG and remove artifacts.

The metrics reported in Tables 6–8 represent *averages across all channels* of each test recording. For each EEG channel, we compute SNR, RRMSE, MI, CC, etc. individually, then report the mean across channels to summarize overall performance. We note two primary observations from a per-channel standpoint.

**Table 8:** Denoising performance of classical baselines vs. GAN-based models. metrics are averaged over all test channels.

**Method**	**SNR**	**RRMSE**	**MI**	**SD**	**MAE**	**PSNR**	**CC**	**DTW distance**
WT Krishnaveni et al. (2006)	9.32	1.011	4.71	3.52	0.135	14.14	0.80	13.98
WF Hermus et al. (2006)	10.05	0.966	4.79	3.47	0.126	14.87	0.82	12.93
GAN	12.10	0.825	5.01	3.14	0.092	18.94	0.90	10.92
WGAN-NoGP	11.88	0.874	4.99	3.21	0.102	18.31	0.88	11.56
WGAN-GP	13.03	0.908	5.07	3.36	0.108	18.65	0.86	10.43

### 7.2 Analysis of Signal Denoising Results

Tables 6, 7 provide a quantitative comparison of the GAN and WGAN-GP architectures under various signal fidelity metrics. The following subsections detail the performance of each model with respect to SNR, PSNR, error metrics, and measures of signal structure preservation.

#### 7.2.1 Signal-to-noise ratio (SNR) and peak signal-to-noise ratio (PSNR)

A comparison of the SNR and PSNR values indicates that WGAN-GP, although effective at mitigating noise, often produces a lower overall SNR and PSNR than standard GAN. In particular, the highest recorded PSNR for WGAN-GP is 19.28 dB (Figure 4C), while the corresponding peak for GAN is also 19.28 dB (Figure 3A), suggesting that GAN tends to preserve more inherent signal quality. This discrepancy highlights the particular strength of GAN in maintaining both the amplitude and frequency characteristics of the original signal while still reducing noise.

#### 7.2.2 Relative root mean squared error (RRMSE) and mean absolute error (MAE)

With respect to RRMSE and MAE, WGAN-GP generally exhibits slightly higher error values across all tested signals. For instance, while the GAN-based model achieves an RRMSE of 0.818 in Figure 3A, WGAN-GP's best RRMSE remains at 1.063 (Figure 4D). These findings underscore GAN's superior ability to minimize both large and small reconstruction errors, which is consistent with its stronger PSNR and SNR performance.

#### 7.2.3 Mutual information (MI) and standard deviation (SD)

Mutual Information (MI) and the standard deviation (SD) metrics provide insight into the ability of each model to retain the structural and statistical attributes of the original signal. GAN achieves higher MI values (ranging from 4.92 to 5.08) than WGAN-GP, indicating that it generally preserves a larger portion of the original informational content. Although WGAN-GP excels at noise suppression, its higher SD scores suggest that it may introduce additional variance or artifacts, slightly compromising overall fidelity.

#### 7.2.4 Dynamic time warping (DTW) distance

WGAN-GP exhibits higher DTW distances, implying reduced temporal alignment between denoised and true signals. For example, GAN achieves its lowest DTW distance of 7.20 (Figure 3C), compared to WGAN-GP's lowest of 8.85 (Figure 4C). This finding may reflect WGAN-GP's tendency to prioritize stronger noise reduction, occasionally at the expense of detailed temporal fidelity.

### 7.3 WGAN without gradient penalty

To further investigate the effect of gradient-penalty regularization on EEG denoising, we conducted an experiment using a *WGAN without GP* (hereafter, **WGAN-NoGP**). Except for removing the gradient-penalty term in the discriminator's loss, all other hyperparameters and training procedures (including batch size, number of critic steps, and learning rates) matched those used for the standard GAN and WGAN-GP runs.

Table 8 compares the performance of **GAN**, **WGAN-NoGP**, and **WGAN-GP** on the same test data.

As shown in Table 8, **WGAN-NoGP** yields intermediate results compared to the standard GAN and WGAN-GP.

1. *Noise Suppression (SNR, PSNR):* WGAN-GP attains the highest SNR (13.03 dB), suggesting it excels at removing low-level artifacts. In contrast, the WGAN-NoGP model exhibits a lower SNR (11.88 dB), indicating less effective overall noise reduction.
2. *Reconstruction Error (RRMSE, MAE):* WGAN-NoGP's RRMSE (0.874) and MAE (0.102) surpass those of the standard GAN, implying that removing GP can somewhat degrade reconstruction accuracy compared to the baseline GAN setup.
3. *EEG Structure (MI, SD, CC, DTW):* Although WGAN-GP achieves marginally higher SD, it maintains a balanced performance in terms of mutual information and correlation. WGAN-NoGP introduces slightly more variance in the denoised signals and yields a higher DTW distance, suggesting that it offers less precise time-alignment compared to WGAN-GP.

Overall, these findings affirm that gradient-penalty regularization contributes to stabilizing WGAN training and enhances artifact removal in EEG signals, particularly under high-noise conditions. WGAN-NoGP remains a competitive alternative when computational resources or tuning complexity are limited, but its denoising quality tends to lie between that of the standard GAN and WGAN-GP.

### 7.4 Comparison with existing works

In recent years, deep learning methods–particularly GANs–have revolutionized EEG signal enhancement and reconstruction. The present study aligns with these advances by employing a WGAN-GP to improve EEG signal clarity while preserving critical structural details. Compared to conventional approaches such as LMS filtering and wavelet-based thresholding, our methodology leverages the adaptive capacity of GANs to learn complex, nonlinear noise profiles (Goodfellow et al., 2014).

Several contemporary investigations demonstrate the efficacy of GANs in dynamic signal enhancement scenarios. For example, Zhou et al. (2020) introduced a Wireless Signal Enhancement GAN (WSE-GAN), which robustly mitigates channel interference, underscoring the adversarial framework's resilience to time-varying environmental factors. Although originally designed for wireless communication data, WSE-GAN's architecture also provides valuable insights into how GANs may generalize to EEG signals exhibiting comparable noise volatility.

Luo et al. (2020) extended these concepts to EEG signal reconstruction by incorporating a TSF loss within a WGAN architecture, achieving high-fidelity reconstructions via multi-dimensional feature integration. In contrast, our WGAN-GP model focuses primarily on time-domain denoising, thereby simplifying computational overhead while attaining improvements in SNR and Mutual Information. Although we do not adopt the spatial or frequency components of TSF loss, our results confirm that effective denoising can still be achieved with a lower complexity design (Radford et al., 2016; Zhang et al., 2021).

In particular, our WGAN-GP approach outperforms standard GAN models and legacy techniques in SNR, MI, and CC. By trading multi-dimensional coverage for computational efficiency, the model maintains strong accuracy in the temporal domain, showing potential for real-time EEG processing where resources may be constrained.

### 7.5 Comparison with non-GAN baselines

We contextualized our adversarial methods by evaluating two classical denoising strategies commonly employed for EEG:

1. **Wavelet thresholding (WT):** decomposes EEG signals into multiple wavelet levels and applies adaptive thresholding at each scale (Krishnaveni et al., 2006).
2. **Wiener filtering (WF):** minimizes mean squared error under a linear model, often effective for mild to moderate noise (Hermus et al., 2006).

Table 8 provides a concise overview of these baselines, evaluated on the same test subset used for GAN/WGAN experiments. We report the same set of metrics (SNR, PSNR, RRMSE, MAE, MI, SD, CC, DTW) to facilitate direct comparison.

1. **Channel variability:** channels over sensorimotor areas (e.g., C3, C4) often showed slightly higher SNR improvements, possibly due to distinctive patterns of motor-related artifact in the raw signals.
2. **Robustness across channels:** while certain fronto-temporal channels (e.g., F7, T3) contained more ocular and muscle artifacts, the adversarial methods exhibited stable performance across most scalp locations, suggesting that their learned noise models generalize effectively.

Overall, the *aggregate* metrics present a balanced view, whereas channel-specific analyses can yield deeper insight into each method's effectiveness under anatomically varying noise conditions (e.g., ocular vs. muscle artifacts concentrated in frontal or temporal sites).

From Table 8 we observe:

1. *Higher SNR & PSNR:* The adversarial methods (GAN, WGAN-NoGP, WGAN-GP) generally achieve substantially higher SNR/PSNR than both Wavelet Thresholding and Wiener Filtering, reflecting improved noise removal without over-smoothing the signal.
2. *Reduced Errors:* GAN-based approaches exhibit lower RRMSE/MAE than the classical filters, indicating tighter alignment with the ground-truth reference waveforms.
3. *Better Structure Preservation:* Higher MI and CC scores, coupled with reduced DTW distances, confirm that adversarial networks retain essential temporal and statistical features of EEG signals more effectively than wavelet or Wiener approaches.

These baseline comparisons demonstrate that the proposed adversarial frameworks not only match but surpass classical denoising methods across multiple metrics, offering greater adaptability in handling diverse EEG artifacts.

By integrating these additional experiments and analyses, we provide a more comprehensive perspective on EEG denoising with adversarial methods. Specifically:

1. **WGAN-NoGP vs. WGAN-GP:** Removing the gradient penalty reduces denoising stability and can slightly degrade reconstruction fidelity, though it remains more robust than a naive (non-Wasserstein) GAN in many scenarios.
2. **Classical baselines:** wavelet thresholding and Wiener filtering offer moderate improvements but consistently underperform compared to adversarial approaches in both objective metrics (e.g., SNR, PSNR, RRMSE) and downstream MI classification tasks.
3. **Practical gains:** the BCI experiment confirms that denoising improvements directly correlate with higher accuracy in decoding user intentions, emphasizing the practical significance of robust artifact removal.
4. **Clean reference generation:** our transparent methodology for obtaining “clean” EEG references (low-artifact segments + synthetic noise injection) provides a reproducible basis for evaluating and comparing denoising algorithms.
5. **Channel-wise considerations:** averaged metrics capture general trends, but analyzing specific scalp regions can reveal localized artifact dynamics and model strengths.

### 7.6 Practical considerations and limitations

Improving EEG quality has considerable practical implications in real-world tasks such as BCI applications, clinical diagnostics, or neurofeedback systems. By focusing on robust artifact removal, our adversarial methods can enhance the clarity of neural signals, potentially reducing the risk that key neural events are masked by noise. While we concentrate on quantitative metrics (e.g., SNR, PSNR, MAE), future investigations could explore task-specific outcomes (e.g., improved recognition of event-related potentials) to confirm how these denoising gains translate into meaningful performance benefits in actual use cases.

While the standard GAN model excels in preserving signal integrity–exhibiting higher SNR, PSNR, and CC, and lower error rates (e.g., RRMSE, MAE)–WGAN-based approaches may be favorable in scenarios where aggressive noise suppression is paramount. WGAN-GP's architectural constraints offer greater flexibility in attenuating noise but can lead to a slight reduction in fine-grained signal fidelity. In effect, WGAN-GP optimizes for stronger denoising at the expense of minor distortions in the underlying waveform.

In summary, the choice between a standard GAN and WGAN-GP depends on the application's tolerance for signal distortion versus the need for robust noise reduction. If retaining high-fidelity detail is critical–such as in clinical EEG analyses–GAN may be more appropriate. Conversely, in environments where mild signal degradation is acceptable, the WGAN-GP model's capacity for comprehensive noise suppression makes it a compelling alternative.

## 8 Conclusion

This work explored the efficacy of GANs for denoising and reconstructing EEG signals, focusing on both a standard GAN (EEG-GAN) and a WGAN-GP. The experimental findings confirm that both adversarial approaches substantially improve signal clarity and mitigate noise, thus reinforcing their suitability for real-world BCI applications.

Although WGAN-GP excels in suppressing high levels of interference and noise, its aggressive denoising strategy can occasionally compromise fine-grained signal details. Consequently, WGAN-GP emerges as a strong candidate in environments where robust artifact removal takes precedence over signal precision. In contrast, EEG-GAN preserves the original signal structure more faithfully and thus proves advantageous for use cases demanding higher waveform fidelity, such as clinical EEG analysis or nuanced motor-imagery studies.

In general, the two architectures serve complementary functions. EEG-GAN aligns closely with applications requiring meticulous retention of EEG waveforms, whereas WGAN-GP caters to scenarios where strong noise suppression outweighs the need for exact reconstruction. These results highlight the flexibility of GAN-based methods for EEG denoising, positioning them as adaptable tools for both high-fidelity and high-noise contexts. By providing distinct trade-offs between signal fidelity and noise reduction, this study underscores the broader potential of GAN-driven models to address diverse challenges in EEG signal processing.

## Data Availability

Publicly available datasets were analyzed in this study. This data can be found here: https://openneuro.org/datasets/ds004362/versions/1.0.0; https://openneuro.org/datasets/ds004022/versions/1.0.0.

## Footnotes

^1^ http://www.bci2000.org
